# Fitness factors impacting survival of a subsurface bacterium in contaminated groundwater

**DOI:** 10.1093/ismejo/wrae176

**Published:** 2024-09-11

**Authors:** Michael P Thorgersen, Jennifer L Goff, Valentine V Trotter, Farris L Poole II, Adam P Arkin, Adam M Deutschbauer, Michael W W Adams

**Affiliations:** Department of Biochemistry and Molecular Biology, University of Georgia, Athens, GA 30602, United States; Department of Biochemistry and Molecular Biology, University of Georgia, Athens, GA 30602, United States; Environmental Genomics and Systems Biology Division, Lawrence Berkeley National Laboratory, Berkeley, CA 94710, United States; Department of Biochemistry and Molecular Biology, University of Georgia, Athens, GA 30602, United States; Environmental Genomics and Systems Biology Division, Lawrence Berkeley National Laboratory, Berkeley, CA 94710, United States; Department of Bioengineering, University of California, Berkeley, Berkeley, CA 94720, United States; Environmental Genomics and Systems Biology Division, Lawrence Berkeley National Laboratory, Berkeley, CA 94710, United States; Department of Plant and Microbial Biology, University of California, Berkeley, Berkeley, CA 94720, United States; Department of Biochemistry and Molecular Biology, University of Georgia, Athens, GA 30602, United States

**Keywords:** Pantoea, pangenome, survival, fitness, outer membrane, NADH dehydrogenase

## Abstract

Many factors contribute to the ability of a microbial species to persist when encountering complexly contaminated environments, including time of exposure, the nature and concentration of contaminants, availability of nutritional resources, and possession of a combination of appropriate molecular mechanisms needed for survival. Herein we sought to identify genes that are most important for survival of Gram-negative *Enterobacteriaceae* in contaminated groundwater environments containing high concentrations of nitrate and metals using the metal-tolerant Oak Ridge Reservation isolate, *Pantoe*a sp. MT58 (MT58). Survival fitness experiments in which a randomly barcoded transposon insertion (RB-TnSeq) library of MT58 was exposed directly to contaminated Oak Ridge Reservation groundwater samples from across a nitrate and mixed metal contamination plume were used to identify genes important for survival with increasing exposure times and concentrations of contaminants, and availability of a carbon source. Genes involved in controlling and using carbon, encoding transcriptional regulators, and related to Gram-negative outer membrane processes were among those found to be important for survival in contaminated Oak Ridge Reservation groundwater. A comparative genomics analysis of 75 *Pantoea* genus strains allowed us to further separate the survival determinants into core and non-core genes in the *Pantoea* pangenome, revealing insights into the survival of subsurface microorganisms during contaminant plume intrusion.

## Introduction

Subsurface microbial communities are drivers of key steps in global elemental cycles [1]. Anthropogenic contamination of subsurface environments with nitrate and various metals can disrupt these microbial communities, resulting in the loss of species and functional diversity, disrupting normal biogeochemistry cycles that in turn hinder the natural recovery of the contaminated site [2, 3]. Investigations into anthropogenically altered microbial communities often focus on metagenomic data to identify key genomic determinants that enable microorganisms to survive in the contaminated environment [4, 5]. These methods, however, do not consider phenotypic data from microorganisms exposed to the contaminated environment and provide no direct insights into the molecular mechanisms needed for microbes to persist within the altered environment.

**Figure 1:**
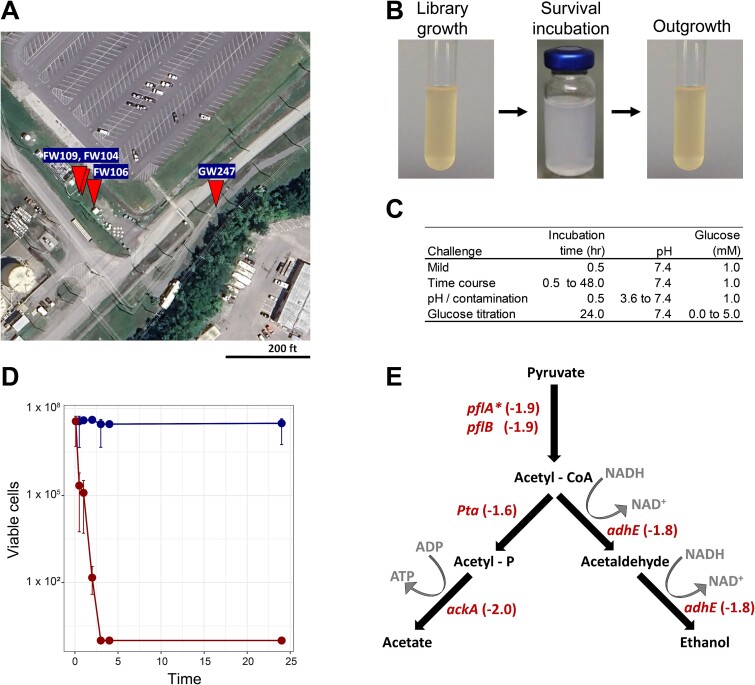
Survival fitness experiments of MT58 in ORR groundwater. (A) Map of ORR S-3 ponds contaminated site in Bear Creek Valley (TN, USA). Markers denote the location of wells within the S-3 ponds contamination plume that were used in this study. Wells used in this study included non-contaminated FW300 (pH 7.4) (not depicted), as well as contaminated wells GW247 (pH 5.4), FW104 (pH 5.0), FW109 (pH 4.4), and FW106 (pH 3.9). Imagery: ©2024 Airbus, Maxer Technologies. (B) Setup of survival fitness experiments with groundwater incubation challenges. (C) Parameters of survival fitness experiments. (D) Survival of wild-type MT58 in non-contaminated (FW300, pH 7.4) and highly contaminated (FW106, pH 3.9) groundwater. (E) Anaerobic pyruvate catabolism in MT58. Values indicate negative fitness values observed under mild groundwater challenge conditions (non-contaminated FW300 groundwater for 30 min). ^*^pflA is an accessory gene required for activation of PflB.

The contamination plume extending from the former S-3 ponds at Oak Ridge Reservation (ORR) in Bear Creek Valley (TN, USA) is an anthropogenically altered site where nitrate and mixed metal contamination affect the subsurface microbial community structure and function (Fig. 1A) [6]. From 1951 to 1983, waste, primarily from uranium processing at the Y-12 Plant and consisting of uranium and various other metals dissolved in nitric acid (pH < 2), was deposited in the S-3 ponds [6, 7]. Despite cleanup efforts, contamination from the ponds seeped into the surrounding environment forming several overlapping plumes [7, 8]. The microbial community structure within these plumes is highly correlated to multiple contamination-related geochemical features, including low pH and high concentrations of metals, including Ni, Co, Mn, Cd, and Pb, as well as U [4, 9]. ORR is a model ecosystem for studying how gradients of environmental stressors impact the composition and function of subsurface microbial communities.

The Gram-negative *Enterobacteriaceae* family is important as it is abundant and widely dispersed in natural and host-associated environments [10]. In particular, the *Pantoea* genus within the *Enterobacteriaceae* consists of versatile and diverse strains with the ability to inhabit wide ranging and dissimilar ecological niches. These include those inhabiting contaminated groundwater and soil, insect symbionts, and important plant pathogens [11]. To a large extent, the survival of a microorganism in a particular environment is dependent on the molecular mechanisms it possesses encoded by the genes in its genome. Consequently, comparative genomics has been used to investigate genomic determinants important for enabling various lifestyles of *Pantoea* species, such as genes associated with onion pathogenicity [12] and coding sequences with orthologs restricted to plant, animal, and insect hosts [12]. In a phylogenomic analysis comparing *Pantoea stewarti* and *Pantoea ananatis* strains, a large core genome of over 3500 shared protein coding sequences was found that was accompanied by a diverse accessory genome composed of genes located on various mobile genetic elements, including plasmids, integrated prophages, and insertion elements [13]. The wide array of mobile genetic element types and the resulting accessory genome supported by the core *Pantoea* genome is likely a key factor in the environmental diversity of this genus. Consequently, the goal of this work was to use a groundwater- and sediment-associated *Pantoea* strain to identify genomic determinants that are key for *Enterobacteriaceae* survival in both non-contaminated and contaminated groundwater environments.

MT58 was isolated from a non-contaminated ORR sediment sample [14]. Despite the fact that MT58 originated from a non-contaminated region, this strain has a high tolerance for both nitrate and various metals (e.g. Cu, Cd, Co, Ni, Mn, U, and Cr) at contaminated site-relevant concentrations [14]. A 16S rRNA gene (V4 region) amplicon sequence variant (ASV) that matches the MT58 16S rRNA gene sequence was detected in ORR groundwater from across the S-3 ponds contamination plume, including highly contaminated samples with pH < 4 and U concentrations >50 μM, suggesting that this ORR *Pantoea* lineage or related *Pantoea* species can persist in the presence of these stressors [14]. MT58 is genetically tractable [15] and reduces nitrate while growing in the presence of elevated concentrations of various metal mixtures, making this strain a relevant model for understanding microbial persistence during anthropogenic nitrate and metal perturbations.

Herein we used survival fitness experiments in which a randomly barcoded transposon insertion (RB-TnSeq) deletion library [16] of MT58 was directly exposed to environmental groundwater samples taken from across the S-3 pond contamination plume. The goal was to identify genes that are critical for survival across a range of groundwater challenge conditions, including exposure time, contamination level, and carbon substrate availability. Additionally, we used comparative genomics to investigate whether any of these “survival” genes are unique to strain MT58, are found in a subset of *Pantoea* species, or are part of the core makeup of the *Pantoea* pangenome. Together, these data allowed us to identify a range of groundwater survival genes from those broadly relevant to Gram-negative *Enterobacteriaceae* to other less widespread accessory genes that are specific to MT58.

## Materials and methods

### Comparative genomics

Complete *Pantoea* genus genomes were obtained from the Bacterial and Viral Bioinformatics Resource Center (BV-BRC) [17]. The rooted phylogenetic species tree was constructed using the KBase [18] Insert Set of Genomes Into SpeciesTree—v2.2.0 app, which uses FastTree 2.0.0 and a set of 49 core genes to infer maximum-likelihood phylogenies [19]. The tree was visualized using Interactive Tree of Life v6.8.1 [20]. Pangenomes were constructed using the mOTUpan—v0.3.2 app [21, 22] in KBase [18] with MMseqs2 easy-cluster mode and 80% minimum coverage for orthologs. Pangenome rarefaction and amplification curves were calculated using a previously published R script with R v4.2.1 [23]. The pangenome openness parameter (γ) was calculated using the equation *G* = *cN*^γ^, where *G* is the pangenome size, *c* is the core genome size, and *N* is the number of genomes [24]. The estimated full pangenome size (*N*_1_) was calculated using the equation *N*_1_ = *N*_obs_ + *F*_1_^2^/2*F*_2_, where *N*_obs_ is the observed pangenome size, *F*_1_ is the number of singletons, and *F*_2_ is the number of doubletons in the pangenome (present in only 1 or 2 genomes, respectively) [24]. MT58 genes and related information were gathered from BV-BRC [17]. Genomic island predictions for MT58 were obtained using IslandViewer 4 [25]. COG categories and annotations for the MT58 genes were obtained using EggNOG-mapper v2.1.5 [26, 27]. Sequence similarity networks for genes of unknown function were constructed using the EFI-Enzyme Similarity Tool and EFI Database v2024_0./100 [28, 29] with the default *E*-value of 10^−5^. The sequence similarity networks were finalized with an alignment score threshold of 35% and by filtering out sequences that deviated from the input sequence by over 20% in length. The sequence similarity networks were visualized using Cytoscape v3.10.2.

### Solutions and media

The 1X salts solution contained 4.7 mM NH_4_Cl, 1.3 mM KCl, 2 mM MgSO_4_, 0.2 mM NaCl, and 0.1 mM CaCl_2_. *Pantoea* minimal medium contained 1X salt solution, 5 mM NaH_2_PO_4_, 1 mM glucose, and a 1X trace element solution described previously [15]. All solutions and media were filter sterilized (0.22 μM) before use.

### Survival experiments

The MT58 RB-TnSeq library [16] was grown on Luria–Bertani (LB) broth [30] with 50 μg/ml kanamycin (Kn) (50 ml) aerobically with shaking (150 rpm) at 23°C to an OD_650_ of 0.5. Six reference samples of the LB culture (1 ml) were harvested, and pellets were frozen at −80°C. The remaining LB culture was harvested (10 min, 6000 × *g*), and the supernatant was decanted. The pellet was washed once with 20 ml of 1X salts and then suspended in 3.5 ml 1X salts. Anoxic (80% N_2_, 20% CO_2_) survival challenge vials containing 3 ml of either *Pantoea* minimal medium or ORR groundwater (filter sterilized, 0.22 μM) with the indicated additions were prepared in quintuplet and inoculated with 100 μl of washed cell suspension (final OD_650_ of ~0.2). Survival challenge vials were then incubated with gentle rocking at 23°C for the indicated amount of time. After the incubation, 100 μl from each survival challenge vial was used to inoculate 5 ml of LB Kn (50 μg/ml) broth for outgrowth (20 hr) aerobically with shaking (150 rpm) at 23°C. The final OD_650_ of the outgrowth cultures was recorded, and 1 ml samples were harvested, and the pellets were frozen at −80°C as outgrowth samples.

### DNA extraction, sequencing, and fitness analysis

Frozen reference and outgrowth sample pellets were processed for DNA extraction and sequencing using the BarSeq98 method as previously described [16]. Briefly, we PCR-amplified the barcodes from the population using a pair of dual-indexed primers that contain all of the adapter sequences necessary for sequencing. The primer pairs all have unique dual indexes to identify instances of index hopping, and the unique indexes allowed us to multiplex hundreds of BarSeq samples on a single lane. After PCR amplification, we mixed these amplicons together in equal volumes, and purified the mixture over a Zymo clean and concentrator 5 column. These PCR amplicons were sequenced using BarSeqV3 primers on the HiSeq2000 platform (Illumina) [31]. Strain fitness values, the normalized log_2_ ratio of counts between the outgrowth and reference samples, were calculated for each bar-coded strain in the library, and gene fitness values, the weighted averages of the strain fitness values for each gene, were calculated as previously described [16]. Quality control and normalization of gene fitness values were performed as previously reported [16]. For the largest gene fitness values and largest gene fitness value changes observed in the survival fitness experiments, a cutoff of ≥|1.5| was selected, which was previously seen to highlight significance results for the MT58 RB-TnSeq library [15]. In cases where gene fitness changes were monitored over a time, pH or glucose concentration, large fitness changes were additionally filtered by fitting the gene fitness value curves to a second-order polynomial and removing genes with poor fits (*R*^2^ value <0.50).

### Viable cell quantification

Viable cells over time were quantified from survival challenge vials prepared as described above. At indicated time points during a challenge, 20 μl of cells were removed from the survival challenge vial and serially diluted 1–10 across the columns of a 96-well plate into LB medium. The plate was incubated for 20 hr aerobically at 23°C with gentle rocking before reading the final OD_650_. The number of viable cells was then determined from the final OD_650_ of the most serially diluted well with growth by comparing the results to those of similarly treated cultures of MT58 with known viable cell counts. Viable cell counts for the MT58 cultures were determined using traditional dilution plating techniques on LB agar plates grown at 23°C for 20 hr.

### Element and ion quantification

Concentrations of 17 different elements were determined using ICP-MS as described previously [32]. Nitrate concentrations were previously determined using a Dionex 2100 system with an AS9 column (U.S. EPA Methods 300.1 and 317.0) [9]. Ammonia concentrations were determined using the Amplite Colorimetric Ammonia Quantitation Kit *Blue Color* (AAT Bioquest, Pleasanton, CA, USA).

## Results

### Survival fitness experimental setup

Five ORR groundwater samples ranging in pH from 3.9 to 7.4 with varying degrees of nitrate and metal contamination from the S-3 ponds contamination source were selected. Sample FW300 (pH 7.4) was from a non-contaminated ORR site located 7000 m from the S-3 ponds, whereas the other samples were taken from wells within the contamination plume (20–40 m distant) (Fig. 1A). The concentrations of contaminating metals (Fig. S1) and other elements/compounds of interest (Fig. S2) were measured. Although most of the metals tend to decrease in concentration with pH, Mo concentration increases (Fig. S1).

Survival fitness experiments were used to identify molecular mechanisms important for microbial persistence during diverse groundwater challenges (Fig. 1B). These experiments involved the use of a RB-TnSeq library in which transposon mutagenesis with constructs containing random DNA bar codes enabled high throughput fitness profiling experiments compared to traditional TnSeq experiments [16]. Each survival fitness experiment was started with MT58 RB-TnSeq library cells harvested from aerobic LB cultures, the growth condition used to construct the RB-TnSeq library [33]. Cells were then resuspended directly into the challenge conditions. These included non-contaminated groundwater, a time course in non-contaminated groundwater, challenges with groundwater samples containing increasing amounts of contamination, and non-contaminated groundwater samples with increasing concentrations of glucose added as a carbon source (Fig. 1C). All challenge conditions were conducted anoxically at 23°C (original state of the groundwater samples), and all except for the glucose titration experiment contained 1 mM glucose. Additionally, a set of challenges was run using the *Pantoea* minimal medium adjusted to the same pH value as the ORR groundwater samples to evaluate differences in fitness observed with pH changes alone rather than exposure to multiple contaminants. After incubation in the challenge conditions for the indicated time periods, the challenge samples were sub cultured back into the original aerobic LB growth condition for outgrowth and fitness analysis. The resulting fitness changes reflect the survival of the library members during the groundwater challenge incubation step. A negative change in gene fitness denotes that the challenge resulted in decreased abundance of library mutants lacking that gene. Survival of the wild-type MT58 strain over time in ORR groundwater with 1 mM glucose added was used to determine limits for the challenge incubation times. Strain MT58 was able to survive without significant decrease in population for 24 hr in non-contaminated FW300 groundwater (pH 7.4). In contrast, a significant decrease in cell viability occurred within 30 min of incubation in the most extremely contaminated FW106 groundwater sample (pH 3.9), with complete loss of viability within 3 hr (Fig. 1D).

### Transition to anaerobic carbon metabolism key to groundwater survival

Even under mild survival incubation challenges in FW300 groundwater (pH 7.4) for 30 min. (Fig. 1C), 35 genes were important for fitness with fitness values ≥|1.5| (Table S1). One of the main changes between the survival incubations and the LB growth condition to which they are compared is the switch to anoxic conditions, a necessary consequence of the experimental design. This approach also mimics an important property of groundwater environments, which undergo changes in O_2_ availability [9, 34]. Consistent with the anoxic environment, the *fnr* gene, which encodes a global transcriptional regulator controlling expression of genes for anaerobic metabolism [35], has a large negative fitness value (−2.3) in the mild challenge. In addition, several genes involved in anaerobic carbon flow that are positively controlled by FNR in *Escherichia coli* also have large negative fitness values (Fig. 1E) [35].

### Stringent response, transcriptional regulators, and outer membrane-related genes important for fitness with increasing time in non-contaminated groundwater

**Figure 2:**
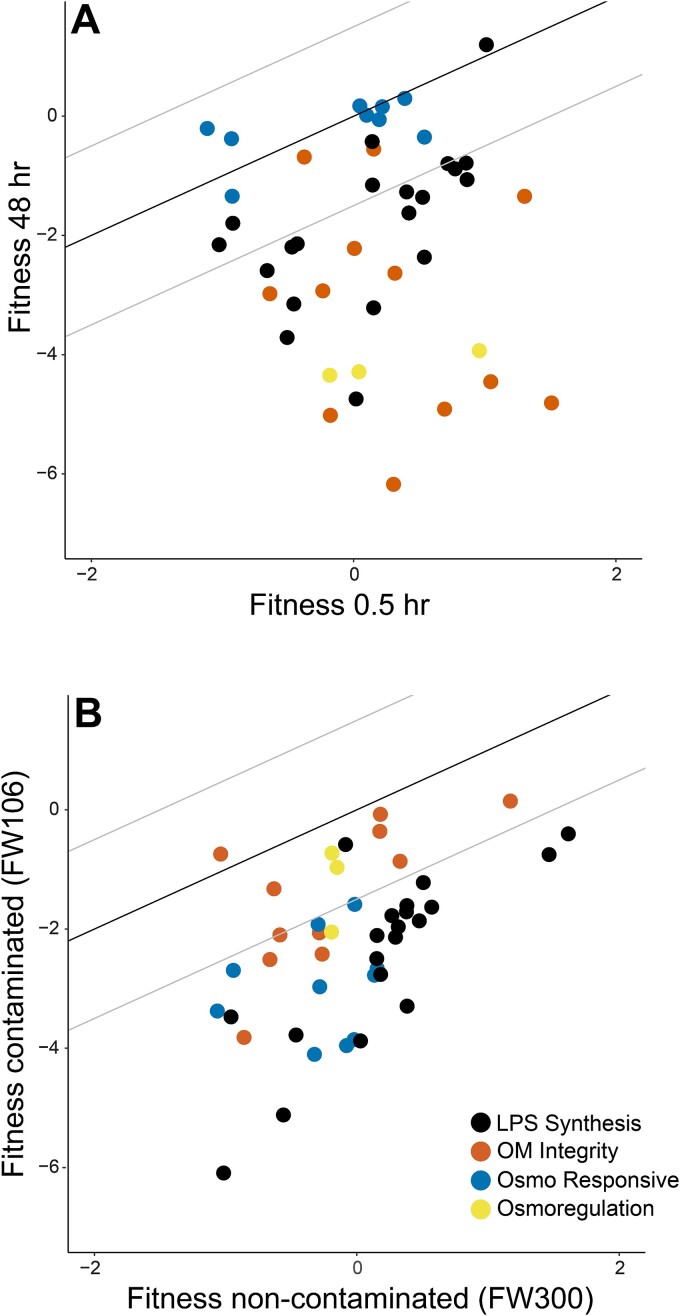
Groupings of outer membrane related genes with similar fitness patterns. (A) Fitness change over time scatter plot after survival incubation challenge in non-contaminated FW300 groundwater for 48 hr versus 0.5 hr. (B) Fitness change with contamination scatter plot after survival incubation challenge for 30 min in contaminated FW106 groundwater versus non-contaminated FW300 groundwater. The black line is the 1:1 line, and the gray lines show the cutoff for large fitness changes at ≥|1.5|. Genes belonging to different categories of outer membrane related genes are detailed in Table S3.

A 48 hr survival fitness time course experiment was conducted in non-contaminated FW300 groundwater supplemented with 1 mM glucose (Fig. 1C). A total of 57 MT58 genes were identified as having large negative fitness changes between the 0.5 and 48 hr timepoints (Δfitness:gw_48h–0.5h_) (Table S2). Several of the genes identified above as being important for fitness in mild conditions (Table S1), had large negative Δfitness_48h–0.5h_ values. Additionally, several genes involved in the stringent response, including *relA* (−3.1), *dksA* (−3.6), and *sspA* (−2.1) [36–38], and several genes encoding transcriptional regulators and transcription factors, had large negative Δfitness:gw_48h–0.5h_ values (Table S2). The latter included *uvrY* (−2.2), which is important for biofilm formation [39], as well as *cspE* (−2.2), part of the RpoS general stress response [40], and *mntR* (−2.2), which regulates Mn homeostasis (Table S2) [41]. Nearly half of the genes with large negative Δfitness_48h–0.5h_ values (28 of 57) are involved in various outer membrane processes (Fig. 2A, Table S3).

### Genes important for fitness in contaminated groundwater include those involved in multiple different outer membrane systems

Survival fitness of the MT58 library was tested in different ORR groundwater samples (supplemented with 1 mM glucose) for 30 min. For comparison, similar survival experiments were also run in the *Pantoea* minimal medium adjusted to the five different pH values of the ORR groundwater samples (Fig. 1C). Only three genes had large negative fitness changes over the pH 7.4–pH 3.9 gradient in minimal medium (Δfitness:mm_pH3.9-pH7.4_) (Table S4). In contrast, there were 45 MT58 genes with large negative fitness changes over the decreasing pH/increasing contamination gradient of the ORR groundwater samples (Δfitness:gw_pH3.9-pH7.4_, Table S4). This includes *sspA*, which encodes a transcriptional regulator critical for acid tolerance in *E. coli* [42], and the Mn efflux gene *mntP* (Fig. S3). As with the time course experiment, most of the genes (32 of 46) with large negative fitness changes with decreasing pH and increasing contamination were related to outer membrane processes (Fig. 2B, Table S3). A total of 20 LPS-related genes had large negative fitness changes with either increased time or increased contamination in groundwater, and 13 of these were important for fitness in both conditions (Table S3). The largest negative fitness change trends for non-LPS outer membrane-related genes generally did not overlap between the time course and increasing contamination experiments (Fig. 2, Table S3).

### Genes involved in aerobic glucose respiration negatively impact fitness in anoxic groundwater

To investigate the metabolic systems critical under different carbon availability regimes, a survival fitness experiment was conducted using non-contaminated FW300 (pH 7.4) groundwater containing increasing amounts of glucose (0–5 mM) for a 24 hr incubation period (Fig. 1C). Several outer membrane integrity-related genes were important for fitness with increasing glucose (Δfitness:glu_5mM–0mM_), including *lpp* (−4.0), *tolQ* (−2.4), *ybiS* (−2.7), and *tolB* (−2.0), as well as the genes *mdoH* (−3.3) and *mdoG* (−3.3), which are involved in osmoregulation (Table S5) [43, 44]. Also, several genes involved in glucose utilization had large negative Δfitness:glu_5mM–1mM_ values, including the glycolytic gene *pgi* (−3.5) and anaerobically-expressed glucose fermentation genes *pflAB* (−1.7, −1.6) and *adhE* (−1.8) (Table S5). By contrast, several genes involved in the aerobic respiration of glucose had large positive Δfitness:glu_5mM–0mM_ values (Table S6). This indicates a competitive advantage for mutant strains in the library that lack functional copies of these genes. Genes with positive Δfitness:glu_5mM–0mM_ values include those encoding subunits of dehydrogenase enzymes in the TCA cycle [45], as well as genes encoding subunits of complex 1 (encoded by the *nuo* genes) and cytochrome o oxidase of the aerobic respiratory chain [46, 47].

Like the genes encoding aerobic respiration, four genes of unknown function also had large positive Δfitness:glu_5mM–0mM_ values with increasing glucose (Table S5). Sequence similarity networks for these genes (Figs S4–S7) revealed related sequences widely distributed in multiple families of the order *Enterobacteriaceae* (*IAI47_RS02400*, *IAI47_RS20625*), multiple orders of the class *Gammaproteobacteria* (*IAI47_RS09550*), and multiple classes of the phylum *Pseudomonadota* (*IAI47_RS05090*). However, none of the sequences in any of the networks were associated with a SwissProt description for a known function.

### *Pantoea* phylogroup containing MT58 enriched in contaminated ORR environment

**Figure 3:**
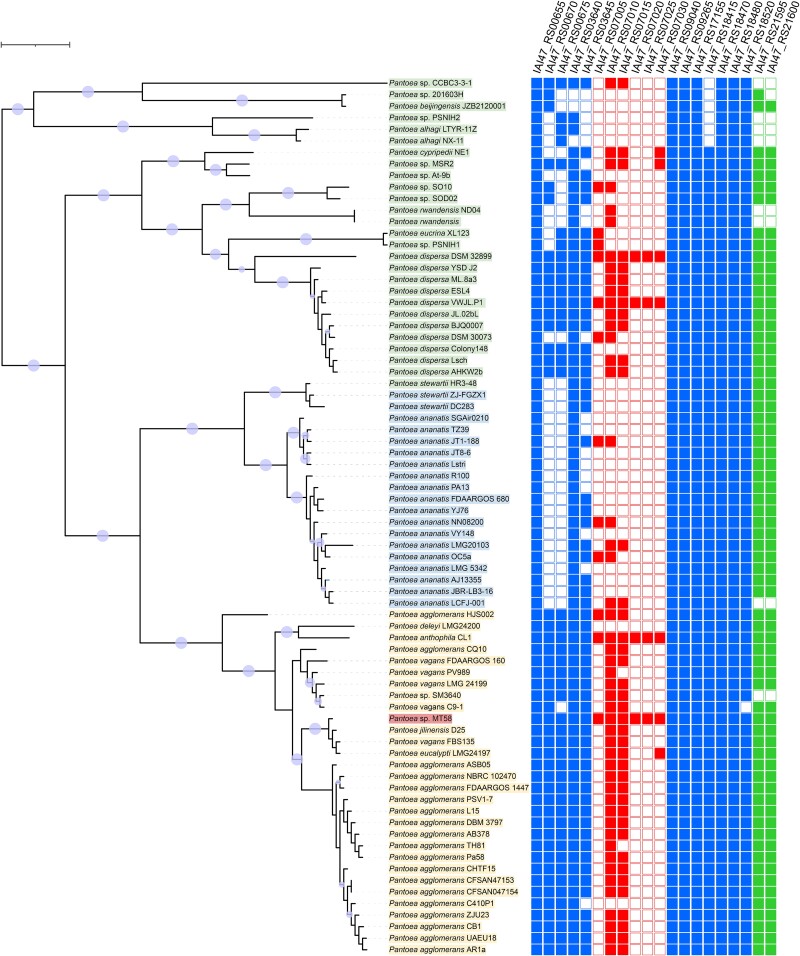
*Pantoea* genus phylogenetic tree. Phylogenetic tree of 75 *Pantoea* genus strains used in this study broken into phylogroups 1 (orange), 2 (blue), and 3 (green). The ORR isolate MT58 is highlighted in red. The tree was constructed using multiple sequence alignments of COGs for 49 core universal genes [19]. Tree scale of 0.01 is indicated in top left corner. Bootstrap values from 0.9 to 1.0 are indicated as circles with larger values having larger circles. The array to the right of the tree consists of genes involved in LPS synthesis that were found to be important for survival in at least one fitness experiment with filled boxes indicating which of the *Pantoea* strains contain a homolog of the MT58 gene. Data was obtained from the *Pantoea* genus pangenome data. The genome location of the gene in MT58 is indicated by color; chromosomal (blue), genomic island (red), and plasmid (green).

We used comparative genomics to contextualize the fitness results with MT58 to determine if the molecular mechanisms important for its survival were a core aspect of the *Pantoea* genus or limited to a subset of *Pantoea* species. Genomes with complete status are available for 75 *Pantoea* strains in the BV-BRC database [17] (Table S7), and phylogenetically, these fall into three main groups (Fig. 3). Phylogroup 1 (30 strains) was mainly composed of *Pantoea* strains of the *Pantoea agglomerans* and *Pantoea vagans* species and includes the ORR isolate MT58. Phylogroup 2 (20 strains) was composed of *Pantoea* strains of the *P. ananatis* and *Pantoea stewartii* species, whereas Phylogroup 3 (25 strains) was more diverse, containing *Pantoea dispersa* strains among others. The majority of the strains were isolated from plant-related sources [42], whereas others were isolated from environmental water and sediment samples [10], from fungi [5], and from various animals, including humans [6] (Table S7).

**Figure 4:**
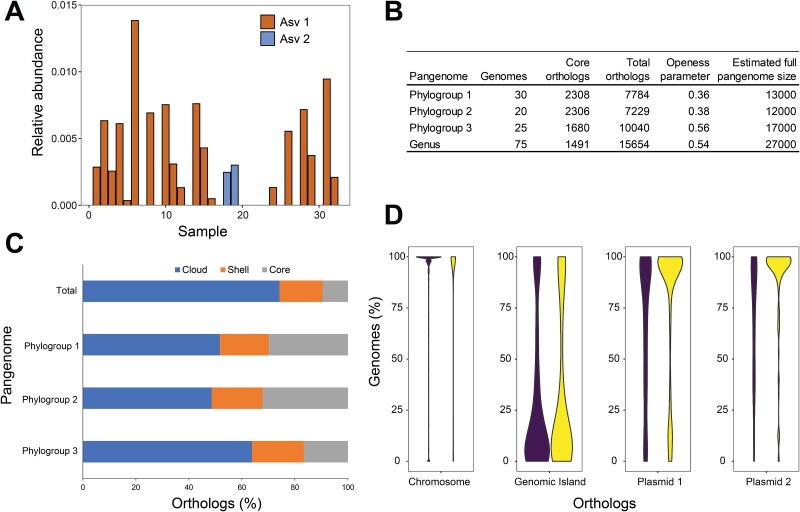
*Pantoea* genus distribution across Oak Ridge Reservation (ORR) contaminated site. (A) Relative abundance of *Pantoea* ASV sequences detected in contaminated ORR sediment samples. Values reported are the highest of two for each sample tested. (B) Summary data for *Pantoea* genus and phylogroup pangenomes. (C) Orthologs located in the core (present in all group strains), shell (within between 15% and 100% of group strains), and cloud (in ≤15% of group strains) of the total and Phylogroup based pangenomes. (D) MT58 ortholog distribution in the *Pantoea* genus and Phylogroup 1 pangenomes by genome location.

The prevalence of various *Pantoea* strains downstream of the S-3 Ponds contamination source was examined using 16S rRNA gene (V4 region) amplicon sequencing data from 32 sediment samples that were previously acquired by cone penetration testing (CPT) [48, 49]. Two *Pantoea* specific ASVs were observed in the CPT samples (ASV1 and ASV2), with ASV1 being present in more samples (19 vs. 2) and at higher relative abundance (Fig. 4A). The more abundant ASV1 was exclusive to Phylogroup 1 and was a 100% match to the rRNA gene sequence of MT58 and to all but two of the other Phylogroup 1 strains. The less abundant ASV2 was a 100% match for all of the Phylogroup 2 strains and a majority of the Phylogroup 3 strains (Table S7). Hence, the extreme ORR environment has a preponderance of Phylogroup 1 *Pantoea* strains.

### *Pantoea* genus and phylogroup pangenomes are open with large accessory genomes

Pangenomes for the 75 *Pantoea* genus strains as well as the three different *Pantoea* phylogroups were calculated (Fig. 4B) [21, 22]. The pangenomes of Phylogroups 1 and 2 had similar percentages of orthologs in the core (present in all group strains), shell (within between 15% and 100% of group strains), and cloud (in ≤15% of group strains) of the pangenomes (Fig. 4C). The Phylogroup 3 and *Pantoea* genus pangenomes had larger percentages of genes in the cloud portion of the pangenome with less in the core. The openness parameter can vary from 0 to 1 and indicates that all four pangenomes are open (Fig. 4B) [24], with higher values being associated with a more open pangenome in which the inclusion of additional genomes to the pangenome is more likely to increase the total number of orthologues. Rarefaction curves for the four pangenomes approach the amount of core genome orthologues and ranged from 1491 to 2308 (Fig. S8). Amplification curves further demonstrate the openness of the pangenomes by not approaching an asymptote (Fig. S8). The estimated size for the four pangenomes ranges from 13 000 to 27 000 orthologues (Fig. 4B), revealing a large accessory genome for the *Pantoea* genus and all three phylogroups.

### Non-core genes and genes located within mobile genetic elements are important for groundwater survival

While most of the genes identified as important for groundwater fitness were part of the core pangenome (Tables S1–S5), next we investigated if there were any metabolic features apparent from the fitness studies that were unique to MT58 or a subset of *Pantoea* species that increase survivability in ORR groundwater. A handful of the genes important for fitness in the time course [6] and increasing contamination [13] groundwater survival experiments (14 total due to overlapping genes) fit this description as they are part of the cloud or low shell (orthologs in <60 *Pantoea* species) of the *Pantoea* genus pangenome (Table S8). Gene locus *IAI47_RS07480* (a hypothetical gene) was the sole MT58 singleton with no orthologs found in the remaining 74 analyzed *Pantoea* species. Three additional genes important for fitness in the increasing contamination experiment were part of the cloud of the *Pantoea* genus pangenome (Table S8). Several of the remaining low-shell genes important for fitness can be described as phylogroup specific. These include four with no representative ortholog in Phylogroup 2 and one (*ydcX*) with no representative in Phylogroup 3 (Table S8). Eight of the 14 subset unique genes are predicted by annotation to be involved in LPS biosynthesis, which we showed was important in both the time course and increasing contamination survival experiments (Fig. 2).

Mobile genetic elements can move genetic information from one organism to another contributing to the organism’s accessory genome. They can be an important factor promoting rapid adaptation to changing environmental stresses. There are several different types of mobile genetic elements known, including extrachromosomal plasmids as well as transposons, integrons, and phages that can integrate into the chromosome [50]. To identify MT58 genes that were potentially acquired from mobile genetic elements, annotation information on each of the MT58 genes was retrieved from the BV-BRC database (Table S9) [17]. The MT58 genome contains three plasmids [pMT58-1 (496 genes, 522 kbp), pMT58-2 (110 genes, 131 kbp, and pMT58-3 (5 genes, 4.3 kbp)] (Table S9). Additionally, we analyzed the MT58 chromosome (3558 genes, 4018 kbp) for integration of other mobile genetic elements using Island Viewer 4.0. This predicted the presence of 19 genomic islands encompassing ~5.7% of the chromosome and 211 genes (Table S9) [25].

**Figure 5:**
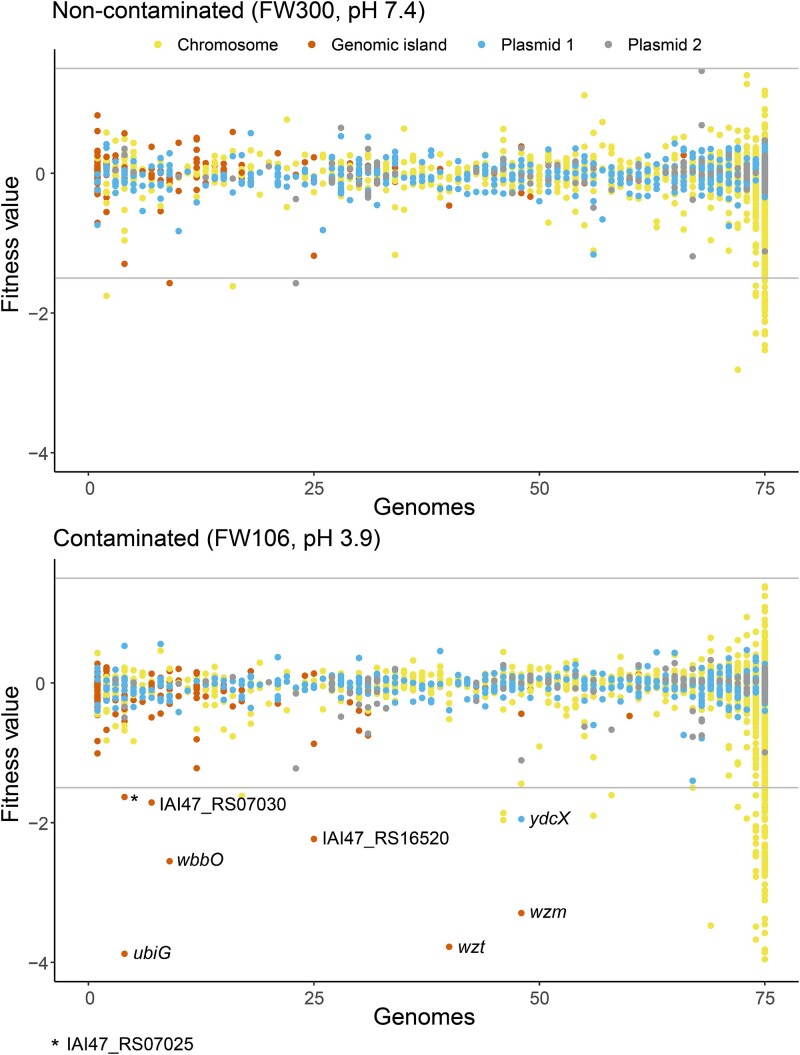
Comparison of gene fitness values after incubation challenge in non-contaminated (FW300) and contaminated (FW106) groundwater. Gene fitness values are from 30-min incubation challenges in the indicated ORR groundwater sample. Gene fitness values are colored based on genome location of the gene and are arrayed on the *x*-axis based on the number of genomes containing an ortholog of the gene in the *Pantoea* genus pangenome. Genes of interest in the low shell or cloud of the Total pangenome (Table S8) are labeled to the right of their corresponding dot.

We divided the MT58 genes into four groups based on their genomic location: encoded on the chromosome, within a genomic island, or on pMT58-1 or pMT58-2. The distribution of chromosomally encoded genes is heavily weighted to the high shell and core of the *Pantoea* pangenome, whereas genomic island and, to a lesser extent, the plasmid-encoded genes are found within the lower shell and cloud of the *Pantoea* genus pangenome (Fig. 4D). The same comparison using the Phylogroup 1 pangenome (of which MT58 is a member) shows a larger proportion of the pMT58-1 and pMT58-2 genes as part of the high shell and core (Fig. 4D). This indicates that these plasmids (or related plasmids) are observed more often in Phylogroup 1 strains compared to the *Pantoea* strains in the other phylogroups. Visualization of gene fitness values in non-contaminated (FW300) and highly contaminated (FW106) groundwater over gene prevalence in the *Pantoea* genus pangenome highlights accessory genes potentially acquired by mobile genetic elements important for contaminated groundwater survival (Fig. 5). A majority of the genes with large negative fitness values in FW106 groundwater that have orthologs in <60 of the 75 *Pantoea* genomes are encoded on plasmids or genomic islands (Fig. 5).

Several plasmid and genomic island associated genes were found to be important in the survival fitness experiments. Three pMT58-1 genes had large fitness changes in the survival experiments. Two were hypothetical genes (*IAI47_RS19945* and *IAI47_RS20625*) that had large fitness changes in the glucose availability experiment (Table S5). The other was *ydcX* in the increasing contamination experiment (Fig. 5, Table S4). This gene encodes the orphan toxin OrtT that was shown in *E. coli* to slow cell growth in response to multiple different stress conditions [51]. Two pMT58-2 genes (*arnE* and *arnF*) were important for fitness in the time course survival experiment (Table S2). The *arnE* gene was also important for fitness in the increasing contamination experiment (Table S4). Both *arnE* and *arnF* are involved in LPS biosynthesis [52]. Six genes of a predicted genomic island on the MT58 chromosome (*IAI47_RS07005* to *IAI47_RS07030*) were important for survival in contaminated groundwater (Fig. 5, Table S4). Within the genomic island, the *wbbO* (−1.7) gene encodes a glycosyl transferase important for O-antigen construction [53]. The O-antigen construction process for *wbbO* is also dependent on an ATP-binding cassette transporter that likely includes *wzm* (−3.7) and *wzt* (−3.3), located within the same genomic island [53]. Two additional hypothetical genes on different genomic islands (*IAI47_RS 07480* and *IAI47_RS 16520*) also had large negative Δfitness:gw_pH3.9-pH7.4_ values (Table S4).

## Discussion

Our *Pantoea* species tree is partitioned into three major phylogroups. Phylogroup 1, which contains MT58, corresponded to the main *Pantoea* ASV found in sediment impacted by the S-3 ponds contamination plume (Fig. 4A). Comparison of the frequency of pMT58-1 and pMT58-2 gene orthologs in the Phylogroup 1 and *Pantoea* genus pangenomes shows that both of these plasmids are enriched in Phylogroup 1 and may be a discriminating factor characteristic of this phylogroup (Fig. 4D). The importance of these plasmids for survival of MT58 in increasingly contaminated groundwater was substantiated with survival fitness experiments that identified one pMT58-1 gene (*ydcX*, −1.7) and one pMT58-2 gene (*arnE*, −2.0) with large negative Δfitness:gw_pH3.9-pH7.4_. Several other Phylogroup 1 specific genes were also found to be important in the time course and increasing contamination fitness experiments (Table S8), supporting the idea that Phylogroup 1 organisms are more prevalent than other *Pantoea* strains in the S-3 ponds contaminated environment due to their specific genomic content. Future efforts to obtain and characterize other *Pantoea* isolates from the ORR contaminated environment could help further define the importance of these fitness-relevant genes and potentially identify others.

The *mntP* and *sspA* genes, important for Mn homeostasis and acid resistance, respectively (Fig. S3), along with 43 other genes, had large negative fitness changes in increasingly contaminated ORR groundwater but negligible changes in minimal medium with decreasing pH (Table S4). Clearly, survival in contaminated ORR groundwater is due to multiple factors in addition to pH, including exposure to toxic nitrate and metals (Figs S1 and S2). An aspect of bacterial survival in the contaminated ORR environment is synergistic effects from multiple challenges. The toxicity of metals can vary greatly with pH due to differences in solubility and oxidation states [54]. In addition, the various ORR metals themselves can have synergistic negative impacts on microorganisms, as was previously observed for a contaminated site relevant ORR *Bacillus cereus* strain [55].

Genes involved in controlling and using carbon and energy sources were among the most important for groundwater survival. Under mild survival conditions, genes involved in the anaerobic catabolism of pyruvate were shown to be important for fitness (Fig. 1E). By contrast, large positive fitness changes with increasing glucose concentrations were observed for genes encoding several enzymes involved in aerobic respiration indicating the function of these genes inhibits survival in anoxic groundwater (Table S6). Although the exact underlying mechanism for this observation is unknown, several possibilities exist. Immediately after transfer from aerobic to anaerobic growth conditions, the TCA cycle and electron transport chain enzymes are still present and active while the terminal electron acceptor O_2_ is absent [56], which could have adverse effects on the redox balance of cellular electron carriers. Active aerobic respiration machinery could also expose MT58 to mechanisms of toxicity in the survival challenges. In any case, the positive fitness phenotype highlights the challenge of a facultative lifestyle in groundwater environments and the importance of the molecular mechanisms that are used to react to changes in groundwater O_2_ concentrations.

One benefit of library-based fitness experiments is gaining phenotypic information on poorly annotated genes [57]. Several genes of unknown function had positive fitness changes with increasing glucose availability, as was seen for genes involving aerobic respiration (Table S5). Like the genes encoding redox enzymes of the TCA cycle and aerobic respiratory chain, these genes of unknown function are part of the core in the *Pantoea* pangenome (Table S5) and are widely distributed phylogenetically (Figs S4–S7). It is intriguing to consider what functions these conserved hypothetical proteins carry out that are possibly related to the well-studied aerobic respiration systems.

In addition to individual genes, fitness experiments can also be useful in uncovering and confirming metabolic connections between sets of genes. A large number of outer membrane-related genes were among the most important for survival in both the time course and increasing contamination experiments (Table S3). Based on annotations (Table S9) and the reported survival fitness trends, several groupings of outer membrane-related genes emerged (Fig. 2, Table S3). Genes involved in maintaining outer membrane integrity were increasingly important for survival in non-contaminated groundwater with increasing time but not increasing contamination (Fig. 2). These genes with high negative Δfitness_48h–0.5h_ values encode several physiologically-related proteins involved in outer membrane integrity and peptidoglycan processing. For example, with corresponding Δfitness_48h–0.5h_ values in parentheses, TolB (−6.5) is a periplasmic protein that interacts with the transmembrane (cytoplasmic) proteins TolQ (−4.8), TolR, and TolA, while also interacting with outer membrane peptidoglycan-associated proteins Lpp (−6.3), OmpA (−2.6), and Pal [58]. Both NlpI (−5.6) and YbiS (−2.7) are involved in the attachment of Lpp to peptidoglycan [59, 60], whereas Skp (−2.3) is a periplasmic chaperone critical in the biogenesis of OmpA [61]. NagA (−2.2), LdcA (−2.9), and Prc (−5.5) are all involved in the synthesis and/or recycling of peptidoglycan (Table S3) [62–64]. Previously, it has been proposed that the role of the Tol-Pal proteins in Gram-negative organisms is to link peptidoglycan to the outer membrane [58, 65]. The similar fitness trends that we observed for multiple peptidoglycan-related genes and the *tolBQ* genes support this idea of an integrated outer membrane integrity system, and clearly this is important for survival in the ORR environment.

Genes involved in LPS synthesis were critical for survival both with increased exposure time in non-contaminated groundwater and with decreasing pH/increasing contamination (Fig. 2, Table S3). LPS is the outermost component of the Gram-negative cell and is critical for outer membrane integrity as well as serving as a permeability barrier [66]. Over 100 genes can be involved in the formation of the LPS layer, which is composed of three parts: the hydrophobic lipid A core, the hydrophilic core polysaccharide, and the O-antigen hydrophilic oligosaccharide [67]. The LPS genes we observed that were critical for groundwater survival are involved in diverse aspects of LPS formation, including lipid A core synthesis (*yrbG*, *rfaG*, and *rfaB*), O-antigen synthesis (*wbnF* and *wecA*), and transport of LPS components across the cytoplasmic membrane (*arnE*, *arnF*, *wzm*, and *wzxE*) [52, 68–71]. LPS-related mutants can be lethal or sensitive to multiple stress conditions related to groundwater survival, including osmotic shock, temperature shock, and exposure to antibiotics and metals [67, 72, 73].

The LPS genes that were important for groundwater survival were of disparate origins within the MT58 genome and *Pantoea* pangenome, even though LPS is a universal molecular mechanism within the *Pantoea* genus and the larger *Enterobacteriaceae* family. Although several of the genes, including *rfaG*, *wzxE*, and *yrbG*, are chromosomally located and part of the core *Pantoea* pangenome, others, like *arnE* and *arnF*, are located on pMT58-2, and still others are part of the pangenome cloud and located within genomic islands (Fig. 3). Some of the chromosomally located LPS genes of interest, such as *IAI47_RS00670* and *IAI47_RS00670*, are also phylogroup specific (Table S3). Taken together, this shows that LPS synthesis genes critical for MT58 survival in groundwater have been acquired throughout the diversification of the *Pantoea* genus via acquisition of multiple different mobile genetic elements (Fig. 3). LPS is often identified as an important factor in microbial pathogenicity, in which modifications can result in host immune system evasion [74]. For example, horizontally transferred LPS genes impact interactions between pathogenic *Xanthomonas* strains and various plant hosts [75]. The data herein suggests that LPS genes acquired from mobile genetic elements are also critical for adaptation in contaminated groundwater environments.

## Conclusion

Microbial communities in anthropogenically altered environments, like the ORR S-3 ponds, often have low species diversity but are enriched in functional genes essential to survival in the contaminated environment [4, 76]. Metagenomic analyses, however, do not provide direct phenotypic insight and typically overlook genes of interest while providing little insight into mechanisms. Survival fitness experiments in which a MT58 RB-TnSeq library was directly exposed to environmental groundwater successfully uncovered genes important for groundwater survival, including outer membrane-related genes pertinent to Gram-negative *Enterobacteriaceae* and carbon catabolic genes pertinent to facultative organisms in general. Contextualization of these results with comparative genomics further revealed a tableau in which a microorganism from a non-contaminated environment relies on a combination of core and accessory metabolic mechanisms for survival when encountering an anthropogenically altered environment.

## Supplementary Material

Survival_Fitness_Supplementary_FIgures_S1-S8_v26_090524_wrae176

Survival_Fitness_Supplementary_Tables_S1-S9_v26_090524_wrae176

## Data Availability

All data generated or analyzed during this study are included in this published article and its supplementary information files.
