# Supplementary material for: Fitness factors impacting survival of a subsurface bacterium in contaminated groundwater
Source: ISME J. 2024 Sep 11;18(1):wrae176. doi: 10.1093/ismejo/wrae176 (PMC11467524; doi:10.1093/ismejo/wrae176)
Supplement: Survival_Fitness_Supplementary_FIgures_S1-S8_v26_090524_wrae176 [file survival_fitness_supplementary_figures_s1-s8_v26_090524_wrae176.pdf]

The ISME Journal

Fitness Factors Impacting Survival of a Subsurface Bacterium in Contaminated Groundwater

Michael P. Thorgersen[1], Jennifer L. Goff[1], Valentine V. Trotter[2], Farris L. Poole II[1], Adam P. Arkin[2], Adam M. Deutschbauer[2], and Michael W. W. Adams[1]

[1]Department of Biochemistry and Molecular Biology, University of Georgia, Athens, GA, USA

[2]Environmental Genomics and Systems Biology Division, Lawrence Berkeley National Laboratory, Berkeley, CA, USA

Supplemental Materials

Figures S1 – S8

Tables S1 – S9

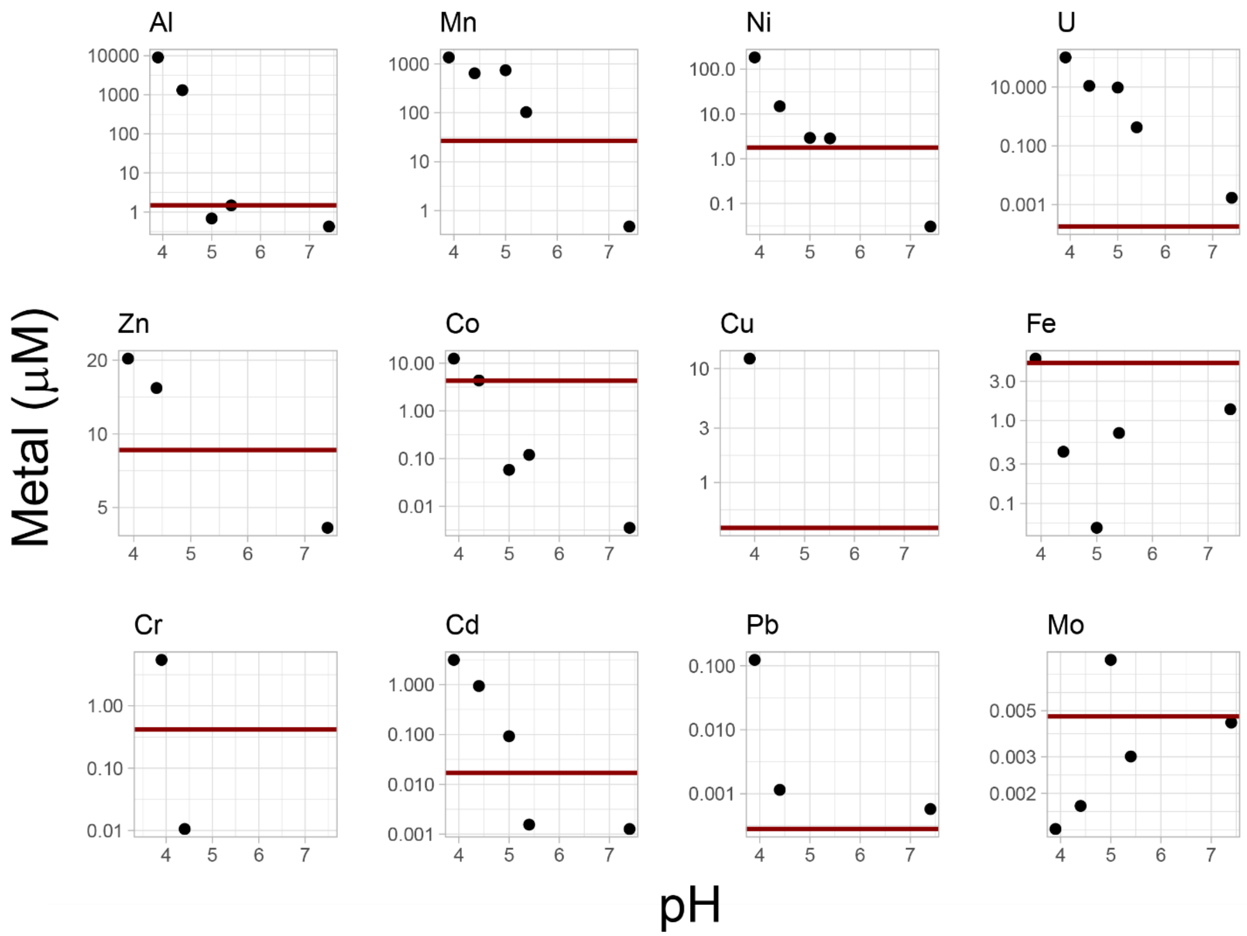

Supplemental Figure 1: Metal concentrations in ORR groundwater samples. The amounts of metals determined by ICP-MS are plotted against pH values of the groundwater samples; FW106 (pH3.9), FW109 (pH4.4), FW104 (pH5.0), GW246 (pH 5.4), and FW300 (pH 7.4). The red line indicates the concentrations of the elements in *Pantoea* minimal medium used throughout the study. In cases where data points are not shown at a particular pH, the value measured was below detection.

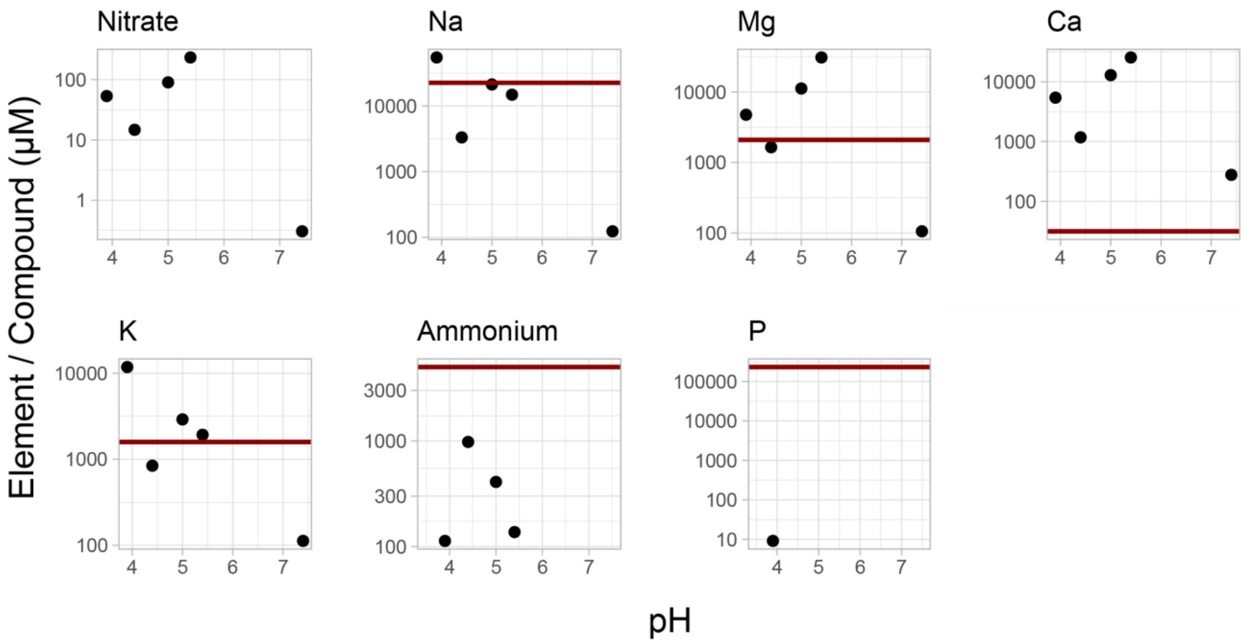

Supplemental Figure 2: Element and compound concentrations in ORR groundwater samples. The amounts of elements determined by ICP-MS are plotted against pH values of the groundwater samples; FW106 (pH3.9), FW109 (pH4.4), FW104 (pH5.0), GW246 (pH 5.4), and FW300 (pH 7.4). The red line indicates the concentrations of the elements in *Pantoea* minimal medium used throughout the study. Nitrate concentration was previously determined [1], and ammonium concentration was determined using a colorimetric enzyme-based kit as described in the methods. In cases where data points are not shown at a particular pH, the value measured was below detection.

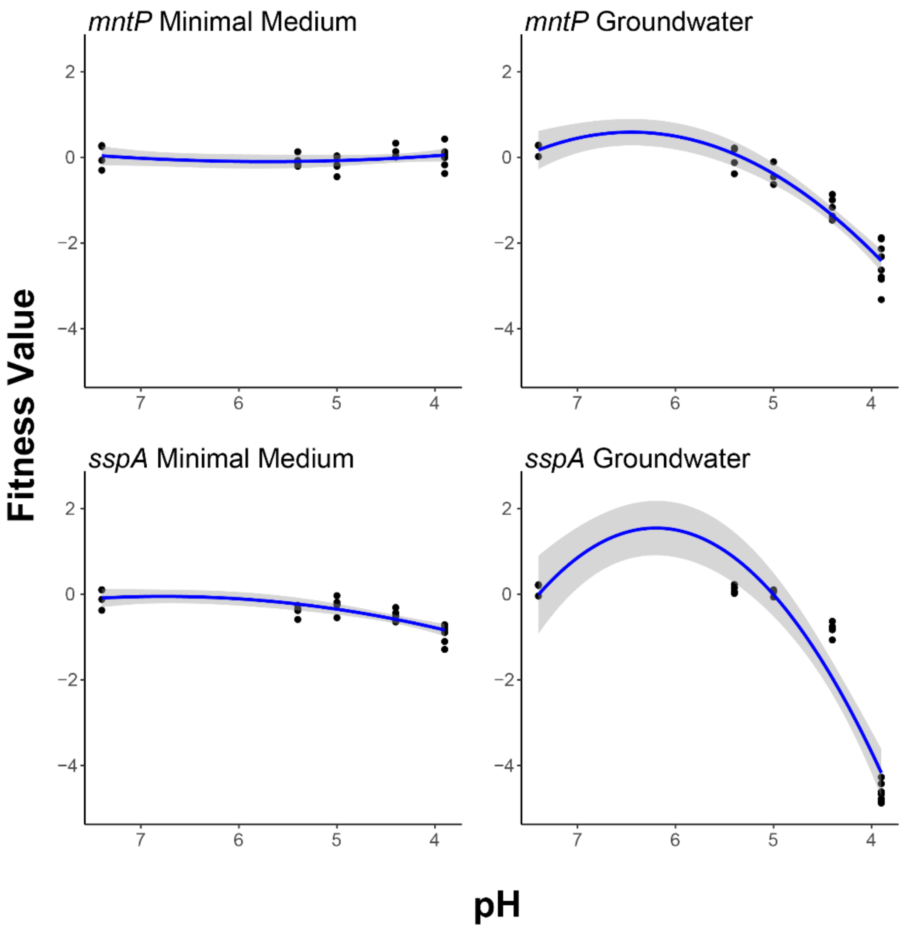

Supplemental Figure 3: Impact of pH and contamination on gene fitness of *mntP* and *sspA.* Gene fitness values are taken from 30-minute challenge incubations of either *Pantoea* minimal medium at different pH values or ORR groundwater samples that had different pH values as well as different metal, element, and compound contamination amounts (Fig. S1, Fig S2). The blue line shows a second order polynomial best fit trendline to the data, and the grey shadow is the confidence interval for the trendline calculated.

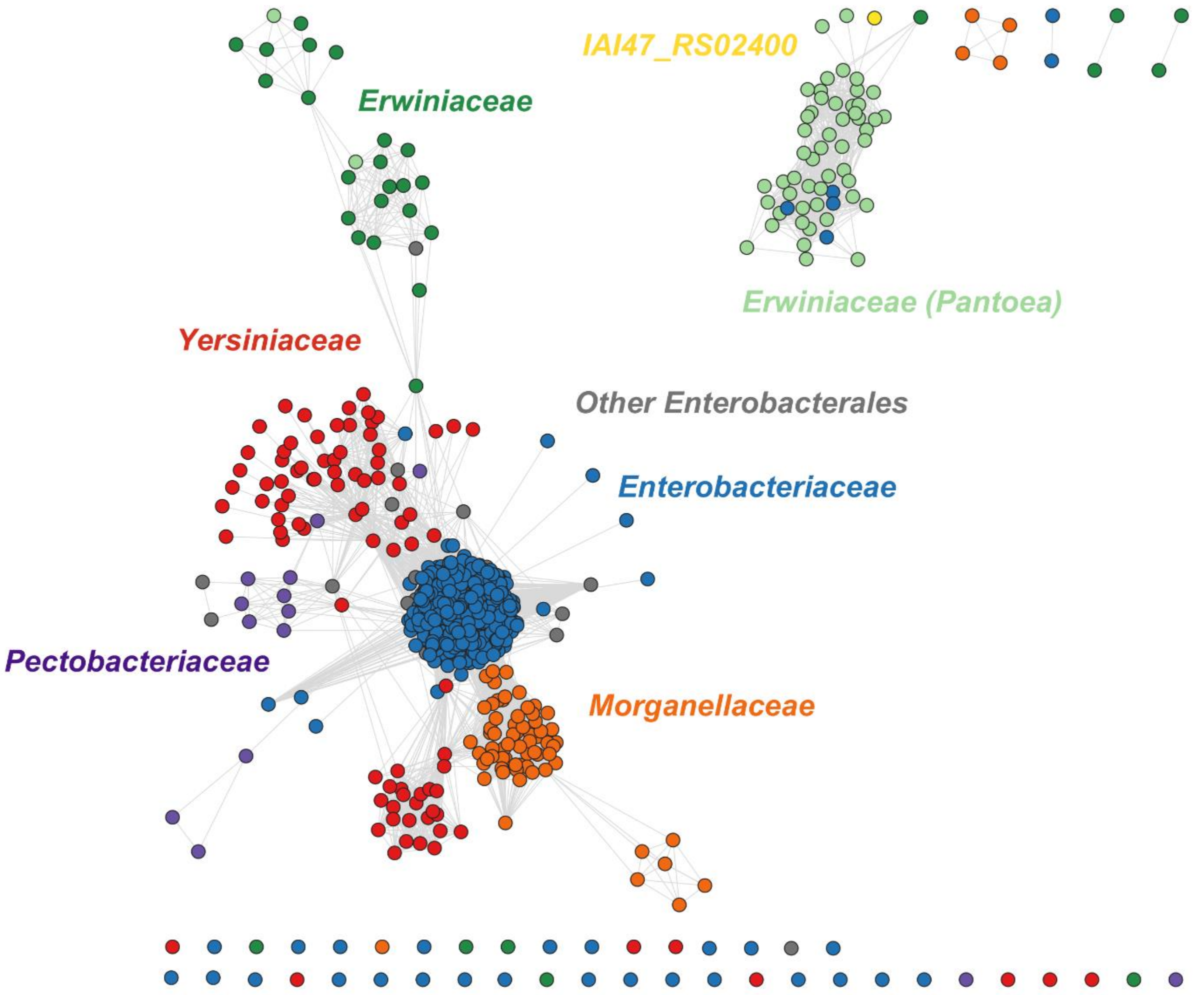

Supplemental Figure 4: Sequence similarity network of *IAI47_RS02400*. The sequence similarity network was generated using the EFI-Enzyme Similarity Tool with the E-value set to the default value of 10^{-5}. The network was finalized trimming out sequences that deviated in length from *IAI47_RS02400* by more than 20% resulting in a network composed of 1,000 nodes and 185,034 edges. Nodes are colored phylogenetically as labeled primarily as different families of the order *Enterobacterales*.

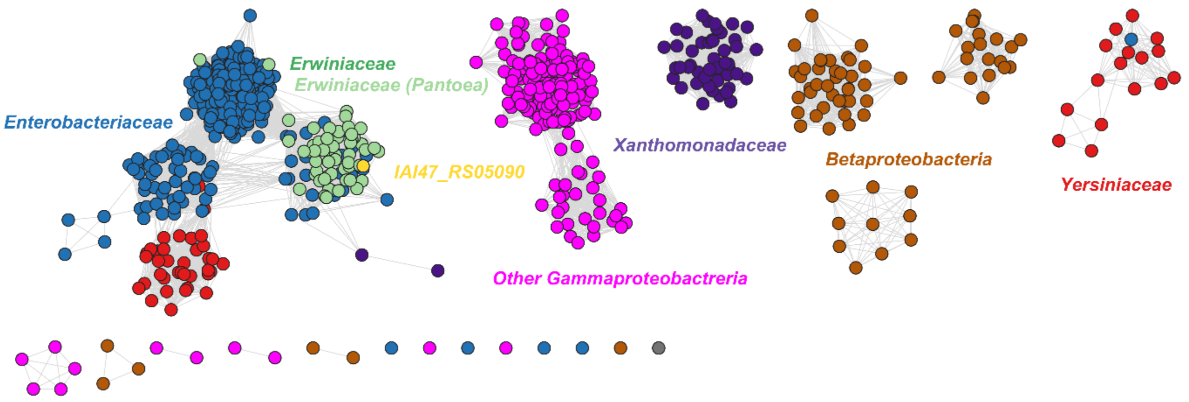

Supplemental Figure 5: Sequence similarity network of *IAI47_RS05090*. The sequence similarity network was generated using the EFI-Enzyme Similarity Tool with the E-value set to the default value of 10^{-5}. The network was finalized trimming out sequences that deviated in length from *IAI47_RS05090* by more than 20% resulting in a network composed of 765 nodes and 46,178 edges. Nodes are colored phylogenetically as labeled primarily as different classes of the phylum Pseudomonadota and different families of the order *Enterobacterales*.

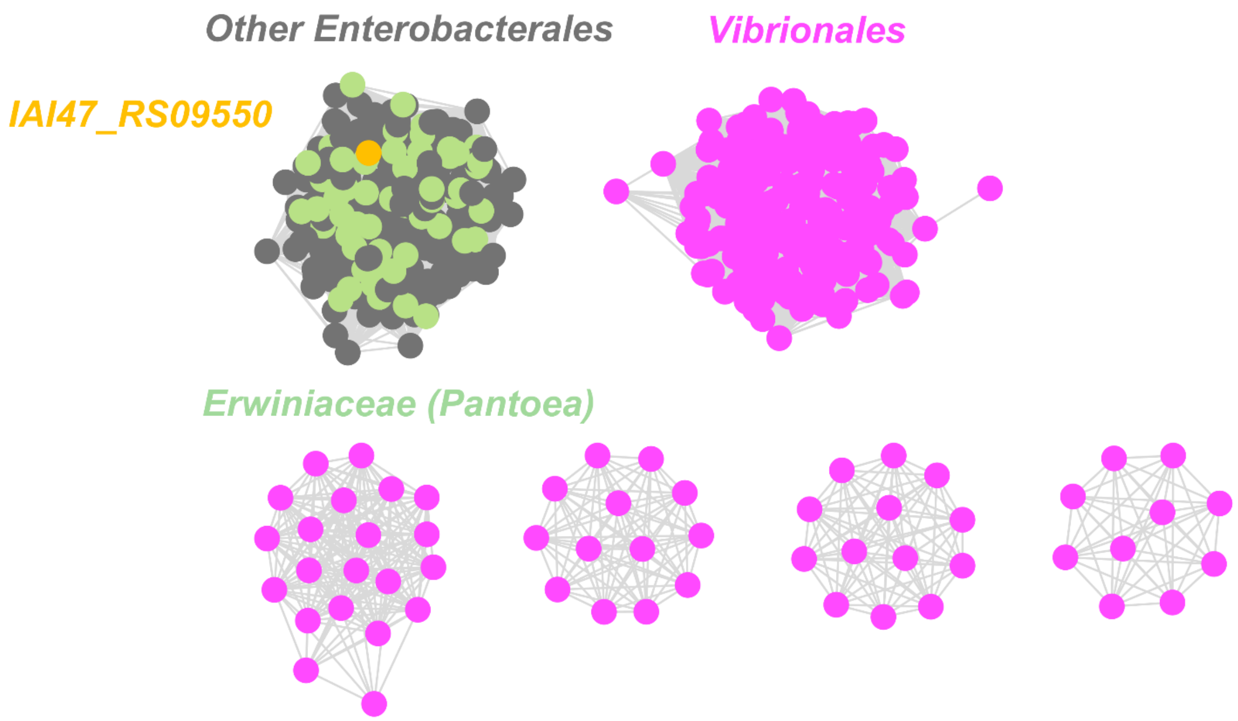

Supplemental Figure 6: Sequence similarity network of *IAI47_RS09550*. The sequence similarity network was generated using the EFI-Enzyme Similarity Tool with the E-value set to the default value of 10^{-5}. The network was finalized trimming out sequences that deviated in length from *IAI47_RS09550* by more than 20% resulting in a network composed of 592 nodes and 58,575 edges. Nodes are colored phylogenetically as labeled primarily as different orders of the class *Gammaproteobacteria*.

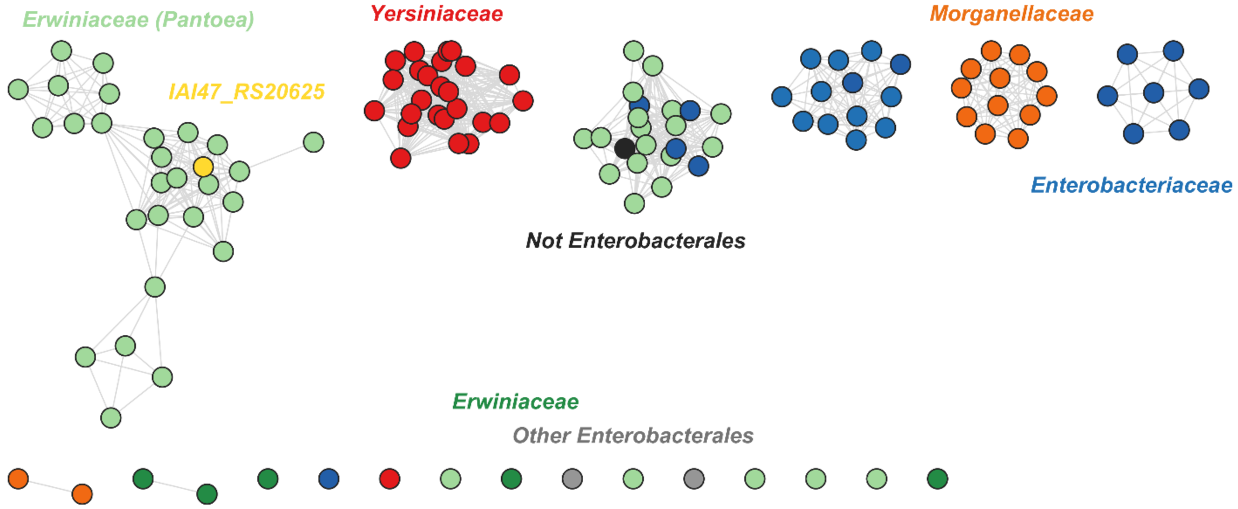

Supplemental Figure 7: Sequence similarity network of *IAI47_RS20625*. The sequence similarity network was generated using the EFI-Enzyme Similarity Tool with the E-value set to the default value of 10^{-5}. The network was finalized trimming out sequences that deviated in length from *IAI47_RS02400* by more than 20% resulting in a network composed of 123 nodes and 761 edges. Nodes are colored phylogenetically as labeled primarily as different families of the order *Enterobacterales*.

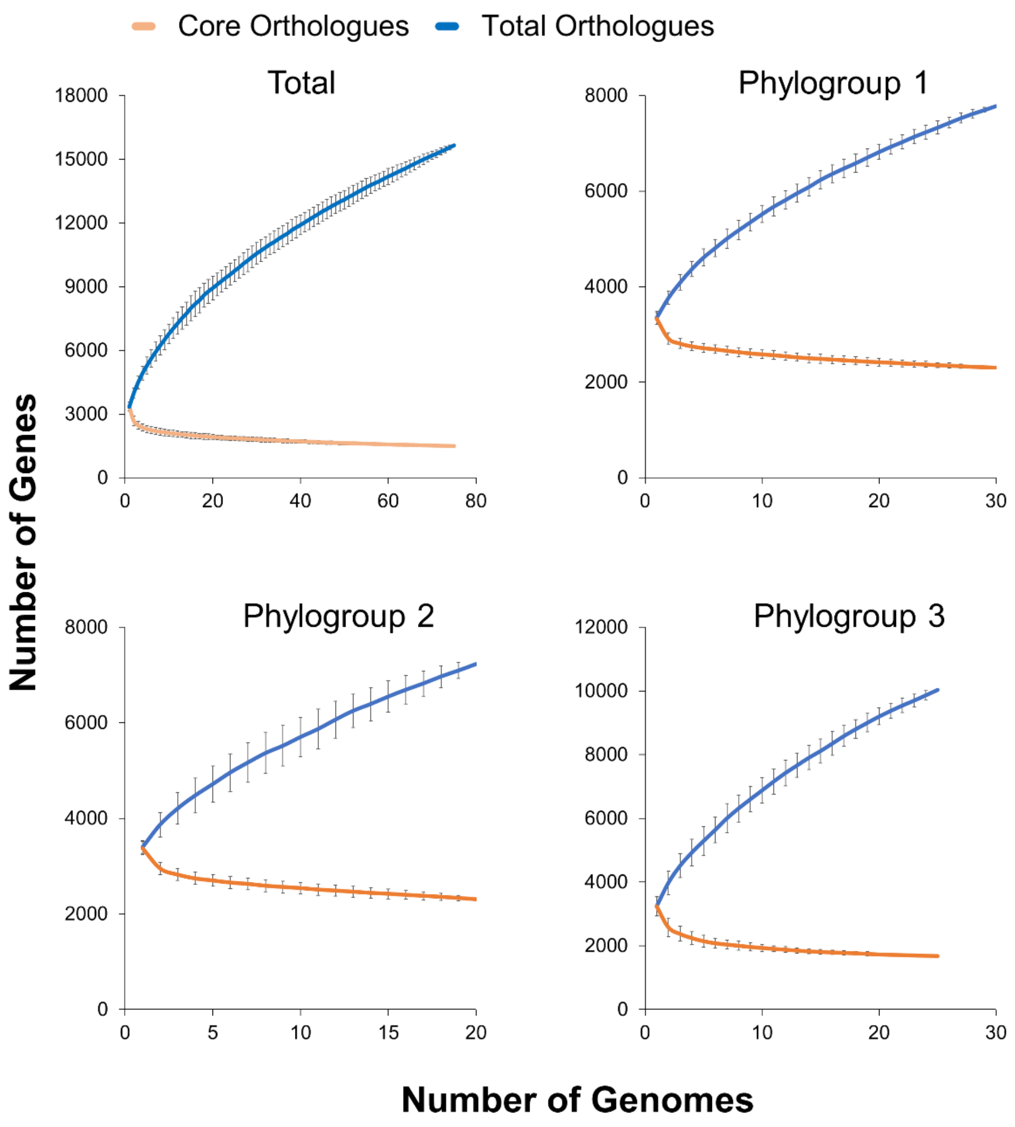

Supplemental Figure 8: Rarefaction and accumulation curves for Total and sub phylogroup pangenomes of *Pantoea* genus strains. The orange and blue lines represent the average core and total genome sizes respectively from 100 repetitions while subsampling an increasing number of genomes. Error bars indicate the standard deviation.

Tables S1 – S9. These are provided in a single excel file.

References

1. Smith, M. B.; Rocha, A. M.; Smillie, C. S.; Olesen, S. W.; Paradis, C.; Wu, L.; Campbell, J. H.; Fortney, J. L.; Mehlhorn, T. L.; Lowe, K. A., Natural bacterial communities serve as quantitative geochemical biosensors. *mBio* **2015,** *6* (3).
